# Hydrometeorological Network of Namal Valley, Pakistan

**DOI:** 10.1038/s41597-025-05310-3

**Published:** 2025-06-11

**Authors:** Muhammad Sheraz, Talha Manzoor, Malik Jahan Khan, Usman Ali, Hamza Tariq, Shabeh ul Hasson

**Affiliations:** 1 Centre for Water Informatics and Technology, Lahore University of Management Sciences (LUMS), Lahore, Pakistan; 2 Department of Computer Science, Lahore University of Management Sciences (LUMS), Lahore, Pakistan; 3 School of Electrical Engineering and Computer Science, National University of Sciences and Technology (NUST), Islamabad, Pakistan; 4 Faculty of Computing, Engineering and Media, De Montfort University, Leicester, LE1 9BH UK; 5 HAREME Lab, Institute of Geography, CEN, Universität Hamburg, Bundesstrasse 55, Hamburg, 20146 Germany

**Keywords:** Water resources, Scientific community, Hydrology

## Abstract

This article describes data collected from a sensor network deployed in the catchment of The Namal Lake, situated in Mianwali district, Pakistan. The lake serves as a reservoir for the Namal Dam. The sensor network consists of 14 unique observation points in a watershed spanning a 425 sq km area. The data includes measurements of precipitation at a resolution of 0.246 mm at all sensor locations and measurements of water level at a 1 mm resolution at 7 locations (including the lake itself). The first sensor installation was made in November 2020 with the last one deployed in July 2022. The associated time series for both variables is generated at a 10-minute sampling frequency. Multiple aggregations of the dataset have been made available at hourly, daily, and monthly timescales. The data can be used for calibrating hydrological models for various applications such as flood prediction, watershed management, and decision-making for reservoir operation. The dataset also contributes to understanding the hydrometeorological processes in similar small catchments at the Monsoon Margin.

## Background & Summary

Small lake systems, often overlooked by research studies in favor of large reservoirs, are now being recognized for their regional and global hydrological importance^1–5^. Studying these systems is challenging due to their large number, remote locations, infeasibility of instrumentation at scale, and limited resolutions of remote-sensing products^6,7^. This is compounded by the strong coupling of hydrological processes and human activities at the catchment level^8,9^. Our study focuses on the Namal Lake system, a reservoir for the Namal Dam^10^ with catchment spanning 425 sq km in the Mianwali district of Punjab, Pakistan. Management of the reservoir involves balancing the competing interests of various stakeholders, for instance, the upstream flood control objective with downstream agricultural water availability. However, the absence of a hydrometeorological observation system, not only prevents effective management of the reservoir, but also increases vulnerability to extreme hydrological events, both of which necessitate accurate on-site measurements^11^. In addition, integrating local hydrometeorological observations into a broader mesoscale meteorological network can significantly enhance the quality and usefulness of environmental monitoring^12,13^. The absence of such integrated networks is evident in the repetitive flood and drought cycles observed in recent decades, which have, at times, contributed to social conflict^14–17^. Addressing the critical need for reliable *in-situ* hydrometeorological observations in such systems^18^, we present the data gathered by a recently deployed sensor network in the formerly ungauged basin.

The Namal valley is surrounded by the Salt Range mountains to the west and south, and agricultural lands to the north and east. The lake is exclusively fed by rainfall with bulk inflows occurring during the Monsoon period (July-September). It is located at the Margin of the Monsoon regime which is reflected in the highly episodic nature of the precipitation patterns. The two main streams, Tarapi and Golar, are the primary contributors, while a third, minor tributary flows into the lake from the nearby village of Rikhi. The lake provides irrigation water to downstream areas via the Namal Canal and also moderates the upstream water table. The dam’s overflow level is at an elevation of 1175 feet above sea level, a threshold that has been surpassed twice since the dam’s construction, specifically in 1976 and 2015^15,17^.

Against this backdrop, a pilot study was initiated to collect high resolution data to support flood prediction and effective reservoir operations. The first sensor was installed in November 2020, to measure lake water level and precipitation. In 2021, the network was expanded with six new stations for precipitation, two of which also record tributary stream levels. Deployments were carried out by the Centre for Water Informatics & Technology (WIT), LUMS. Deployment activities have been presented previously^19^, including the strategy for sensor placement, areal rainfall estimation, and the potential lead-time for warnings of heavy inflows in the Tarapi tributary. Another study^20^ describes an indirect approach to calculating discharges in the streams, with projections from a SWAT-based hydrological model, calibrated by the sensor network data.

Following the completion of the pilot study, a consortium was formed between The Centre for Water Informatics & Technology at LUMS, The NUST School of Electrical Engineering and Computer Science (SEECS), and the Hydro climatology and Remote Sensing of Mountain Environments (HAREME) lab at the Institute for Geography of The University of Hamburg. The activities were supported by the German Academic Exchange Service (DAAD). Under the project, the sensor network was further expanded to a total of 14 locations, with precipitation measurements at all locations and water level measurements at 7 of those locations. The details of the installation locations are given in Table 2.

The project consortium has utilized the data in a range of applications. As mentioned previously, the data has been used to calibrate a hydrological model used to determine discharge in response to rainfall events. The model has been integrated into a Model Predictive Control (MPC) framework to support reservoir operations. Furthermore, the data has been combined with satellite imagery to identify the elevation-area and elevation-volume relations for the lake. These relations are critical for determining water availability in the reservoir. The high temporal resolution of the data makes it suitable for AI-based predictive models which can more accurately represent the episodic nature of the local hydrology compared to conventional frameworks. The data is shared regularly with the local office of the provincial irrigation department (who are responsible for the dam operation and maintenance) and compared with their records. Overall, the data creates an opportunity to uncover critical insights into other small lakes in the Pothohar Plateau, which hosts over 50 small lakes^21^ in a region that contributes to over 5% of Pakistan’s agricultural production^22^.

## Methods

In this section we describe the important features of the data acquisition pipeline, including the device hardware, site selection, sensor maintenance, data processing, and quality control procedures. The relevant guidelines and protocols for each stage have been identified through the reports published by the World Meteorological Organization (WMO)^23–26^.

### Sensors and device hardware

The data is obtained through a sensor network installed across the catchment area of Namal Lake. The devices are prepared indigenously at the Centre for Water Informatics & Technology at LUMS. Relevant applicable guidelines and standards for individual instruments are followed^27^. Here we describe the hardware composition of a single sensor node device. For each node, the device functionalities are maintained by an onboard microcontroller (Microchip PIC18F26K20^28^), that serves as the central control unit and interface with sensors and the data transmission components. Each device collects data at a default sampling frequency of 10 minutes. The data is transmitted to a server using a GSM module (SIM900D^29^) according to a configurable transmission frequency. The transmission frequency is typically set to one transmission per hour, however transmission may fail subject to telecommunication network signal strength. In the event of an unsuccessful transmission, the device temporarily stores the data in its internal memory and appends it to the information being transmitted in the next cycle. In order to enhance operational lifespan, the device harvests energy via a dedicated solar panel to charge an auxiliary battery. To ensure uninterrupted timekeeping, a separate battery powers the Real-Time Clock (RTC). A complete list of the components used are given in Table 1 with further details on the electronics presented elsewhere^30^. The core electronics consisting of the microcontroller, and the GSM module can be interfaced with multiple sensors and has been employed in different configurations in past studies^31–33^. Applicable guidelines for automatic weather stations may be found in the report on observing systems published by the WMO^34^.

**Table 1:** This table provides an inventory of sensors and associated core components employed as part of the device hardware.

Sr. No	Component	Type/Model
1	Water Level	Maxbotix Ultrasonic Sensor MB7380^35^
2	Rainfall	Tipping Bucket Rain Gauge^36^
3	Microcontroller	Microchip PIC18F26K20^28^
4	Wireless Module	SIM900D^29^
5	Antenna	GSM (900 MHz)
6	Solar Panel	1 Watt (5 V)
7	Rechargeable Batteries	Li-Ion 18650 (4.2 V)

The data for the Namal catchment is captured through two separate sensors for water level and precipitation. For water levels, we use an ultrasonic range finding sensor (Maxbotix MB7380^35^). This is used to record water levels at eight different points in the catchment, including the lake elevation and critical points in the stream network (see Table 2). Depending on the location, two versions of the sensor have been used, one with a maximum range of 5 m and the other with a maximum range of 10 m. The minimum range of the sensors is 0.5 m and the measurements have a resolution of 1 mm. For Rainfall, we use a tipping bucket rain gauge (Argent Data Systems^36^) with a resolution of 0.2794 mm. All 14 observation points record precipitation levels as shown in Table 2. While satellite-based precipitation measurements have been employed in past studies of the region^37–39^, no such network of *in-situ* sensors exists, at least to the authors’ knowledge.

**Table 2:** Details of deployed stations in the hydro-meteorological network of the Namal Watershed.

Sr. No	Station Name	Station Type	Latitude (dd)	Longitude (dd)	Altitude (meter)	Deploy Date	Data Gaps (dd-mm-yy)	Current Status
1	Bait-Us-Salam (BS)	R	32.665980	72.026280	552	18-07-22	19-11-23 To 12-05-24	Active
2	Dhoke Kund (DK)	R	32.632624	71.863785	403	29-03-21		Active
3	Dhoke Peera (DP)	R	32.573324	71.890877	653	29-03-21		Active
4	Kalri (KL)	R	32.726692	71.782116	396	15-04-21	28-02-22 To 23-07-22	Active
5	Kund House (KH)	R	32.697785	71.868623	377	29-03-21		Active
6	Narra (NR)	R	32.618286	71.933671	470	19-07-22		Active
7	BudhyWali (BW)	R, WL	32.624114	71.911591	437	04-07-22		Active
8	Dhibba (DB)	R, WL	32.679080	71.873411	374	09-06-21		Active
9	Khebran (KB)	R, WL	32.678199	72.007930	476	04-07-22		Active
10	Lawa (LW)	R, WL	32.692773	71.940513	398	28-03-21		Active
11	Namal Dam (ND)	R, WL	32.665219	71.802257	347	23-11-20		Active
12	Rikhi (RK)	R, WL	32.705030	71.784673	385	04-07-22	09-01-24 To 26-07-24	Active
13	Golarr Bridge (GB)	R, WL	32.639931	71.873194	399	23-06-22	09-12-22 To 30-09-24	Removed
14	Saiflan (SF)	R, WL	32.658041	71.786545	287	04-12-21	28-01-22 To 09-12-22 14-01-23 To 30-09-24	Seasonal

### Sensor network deployment and maintenance

**Fig. 1:**
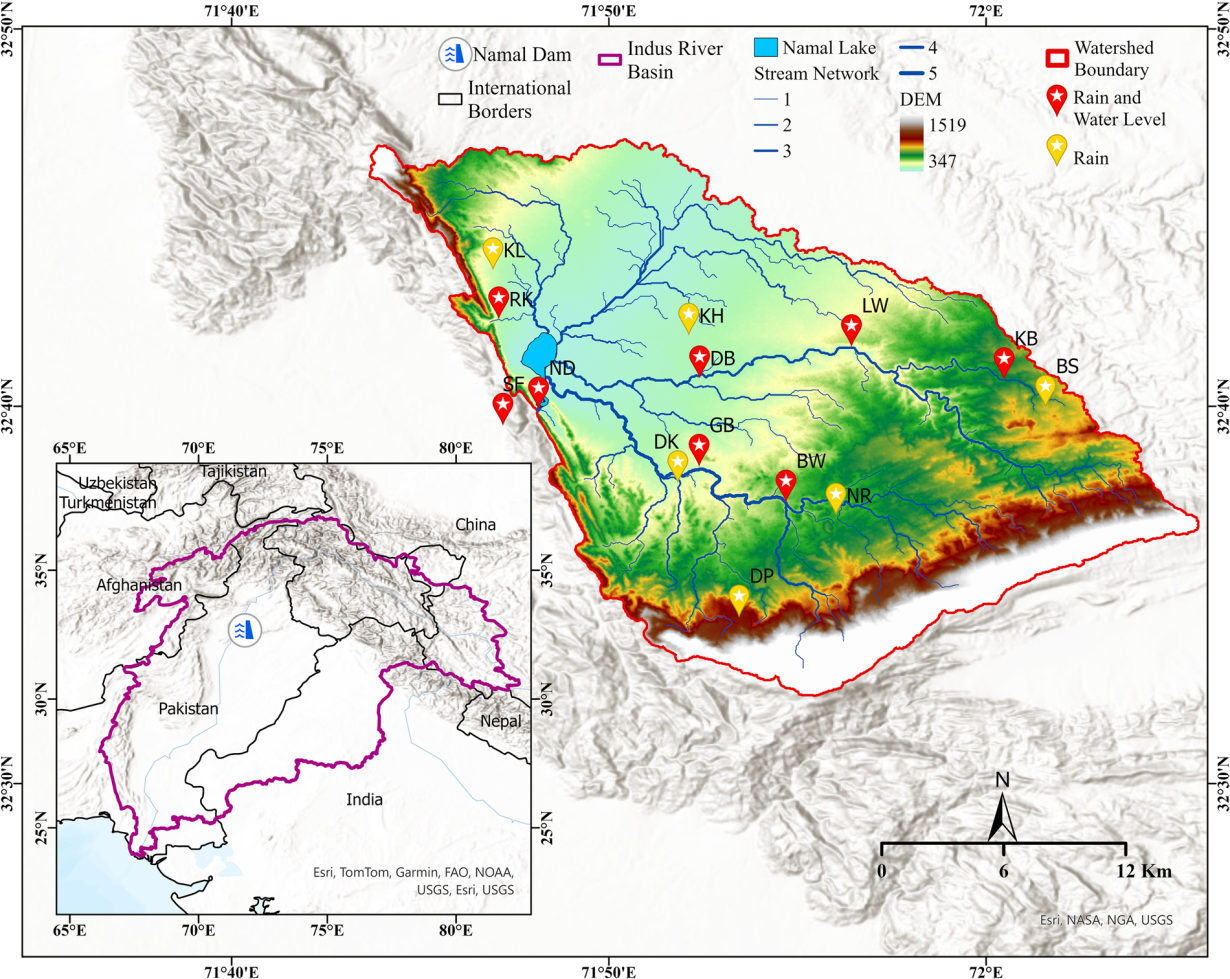
Namal watershed with all observation points associated with the deployed sensor network. The location of the Namal catchment in relation to the Indus Basin is also shown.

Table 2 summarizes the details of our sensor network, initiated in November 2020 with the deployment of the first node at Namal Dam. Apart from the device locations, other critical information including installation dates, device labels, type of measurement, geographical coordinates, current status, etc. are also mentioned. After the first deployment at the dam, the network has progressively expanded throughout 2021-2022 with the addition of rainfall and water level measuring devices made at strategic locations. The locations for the sensors have been chosen strategically. For the water level sensors, there is less flexibility since the sensors are constrained to be located on the respective stream/lake in question. The location of the rain gauges associated with these sensors are thus pre-determined through the field surveys. For the remaining rain gauges, sensor nodes are placed to optimize an information-theoretic criterion over a distribution of past rainfall. More specifically, initial locations falling in the catchment area were obtained from 27 grid points covered by the PERSIANN-CCS rainfall dataset. After eliminating physically inaccessible sites, pairwise covariances were obtained through historic rainfall values. Starting with the predetermined rain gauge locations attached with the water level sensors, the locations for the additional rain gauges were then selected to maximize the Mutual Information criterion between gauged and ungauged locations. A near-optimal procedure was adopted following a greedy algorithmic approach^40^. The obtained locations were then adjusted based on the nearest available site feasible for sensor deployment. Major factors considered include willingness of property owners to allow sensor placement, complexity of the mounting pole required (for stream sensors), accessibility for sensor maintenance, and protection from tampering of the sensor. Since the devices broadcast their data over cellular mobile network, the signal strength of the area was also considered. We discuss complete details of the procedure in a previous study^41^ (also see^24^ for the general guidelines on sensor site selection). The final sensor locations can be seen in Fig. 1. Note that the north-east area of the catchment is not covered by the monitoring network. The stream depicted in that area is derived from the Digital Elevation Model (DEM) and appears only due to the topographic delineation. However, the area remains dry throughout the year, and there is an extremely low contribution from this region to the lake. Constrained with limited budget, the network thus prioritizes catchment regions with significant rainfall that also contribute significantly to the lake water.

**Fig. 2:**
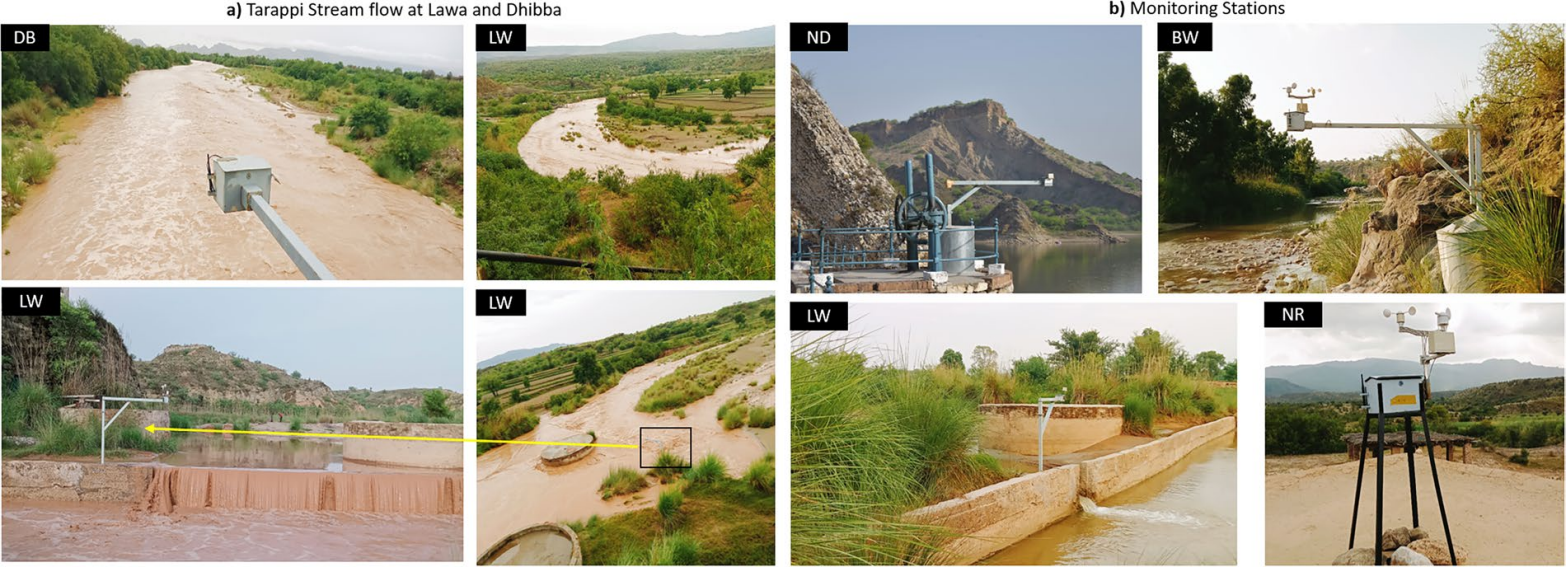
(**a**) Tarapi stream flows at Lawa and Dhibba station, and (**b**) Images showcasing a few of our network’s monitoring stations.

Figure 2 includes pictures of sensor nodes at some selected locations. For sites where a bridge is situated (see the sensor at Dhibba (DB) in Fig. 2a), the sensor pole can be mounted in a relatively straightforward manner by attaching it to the bridge railings. At Lawa (LW), the sensor is securely fastened to a check structure by welding it to bolted plates attached to the structure itself. Due to the direct exposure of the sensor to stream inflow at locations such as at BudhyWali (BW) (Fig. 2b), concrete-filled steel drums were used to secure the mounting poles. The same mounting structure was implemented for the dam (Fig. 2b) to avoid the vibrations caused by implementing other structures that require heavy drilling. Monitoring stations consisting only of rain-gauges were placed on rooftops of houses owned by local community members.

From Table 2 we see that some stations are not continuously kept operational. Specifically, the Saiflan (SF) station, located on the spillway, is deployed on a seasonal basis, capturing data only when dam gates are open in the winter. On the other hand, the Golarr Bridge (GB) station was permanently removed after the theft of a unit from its remote bridge location.

**Fig. 3:**
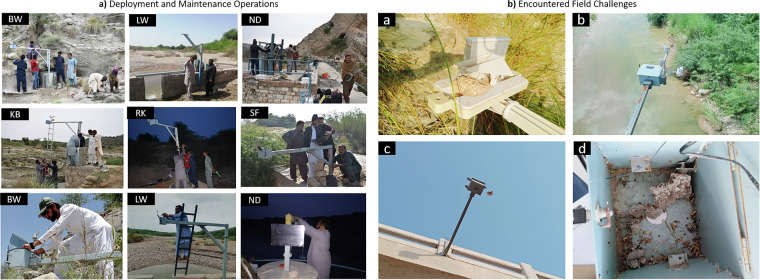
(**a**) Deployment and Maintenance Operations: The top two rows depict the installation of sensors at various locations across the Namal Valley network, showcasing different site conditions and deployment setups. The bottom row highlights the maintenance operations. Labels depicting the station names have been mentioned in the top left corner of each image. (All identifiable individuals in the figures have provided their consent for publication). (**b**) Field Challenges Encountered: These pictures depict various challenges encountered during fieldwork, including: b-a) The presence of a mud dauber nest at BudhyWali Unit, b-b) tampering with out-of-the-box sensors at Golarr Bridge, b-c) tampering with out-of-the-box sensors at Dhibba Karsiyal Bridge, and b-d) a notable wasp infestation inside the Dam Unit.

Proactive maintenance is essential to ensure the reliability of our monitoring network. A local field engineer conducts regular visits to each node to assess the physical state of the stations and to perform maintenance activities. Figure 3b shows some of the encountered field challenges such as spider webs, accumulation of mud, and general wear and tear that can impact the sensor readings. At times, the batteries may also need replacement due to a malfunction in the solar charging component. The frequency of site visits is set according to the criticality of the monitoring station. For instance, the Namal dam station is a high priority unit. If a system failure is detected, the field engineer attempts to resolve the issue onsite, and if unsuccessful, the units are taken to the lab for repair and subsequent redeployment. All maintenance activities are planned to minimize disruptions to data collection and to ensure timely restoration of network functionality in the event of device malfunction.

### Data collection and processing

Following transmission from the monitoring devices to the server, the raw data undergoes a preprocessing and filtering routine, in line with the applicable standard procedures and guidelines^42^. Data is downloaded from the server on a monthly basis and appended to the dataset. The first action is to remove any data reported while the device was removed from the mount during regular field maintenance or testing of the hardware. Next, an automated script removes any garbage data arising from corruption of the communication channel. The raw values reported by the sensors are then converted into physical units. For instance, the raw data from the rain gauges give the number of tips of the tipping bucket as cumulative “ticks” since 12:00 am on the date in question. A conversion factor is then applied to convert the ticks into millimeters. Afterwards, a series of checks is applied to eliminate specific error, for instance, after the reset time, the cumulative rainfall data must be non-decreasing, and any reading that violates this trend is removed. Finally, the absolute values of the data in physical units must obey physical limits imposed by the nature of the processes being observed^26^. This is ensured by removing data that does not obey certain predefined thresholds based on site-specific plausible values. These thresholds, detailed in Table 3, correspond to the physical conditions observed at the installation points. For water levels, the maximum threshold levels correspond to the distance of the sensor to the lake or stream bed, and the minimum threshold levels correspond to the maximum possible historical level of the water (as described by official records or local community members) after incorporating some margin of safety. For the rain gauges, each rain event is confirmed individually from local residents situated at the observation sites. Each instance where a false rain event is detected usually corresponds to a physical malfunction (see Fig. 3b). For each such instance, the gauge is visited to confirm this and serviced accordingly. The threshold for the gauges is set in order to remove all instances of such false events with unusually high rain detected.

**Table 3:** Thresholds used for quality control procedures of the hydrometeorological data, with the maximum and minimum values.

Variable	Max (mm)	Min (mm)
10-minutes Precipitation	30	0
+ daily Precipitation	100	0
Lawa (LW) Water Level	3500	0
Saiflan (SF) Water Level	3500	550
BudhyWali (BW) Water Level	3500	550
Rikhi (RK) Water Level	3500	1000
Khebran (KB) Water Level	4000	1000
Dhibba (DB) Water Level	7000	3000
Namal Dam (ND) Water Level	9500	5000

The ultrasound sensor reports its distance to the water surface in millimeters. The thresholds in Table 3 are specific to each observation point, after incorporating the distance of the sensor to the stream bed and the perceived maximum water level with a margin of safety factored in. To finally obtain the water level in stream locations, the raw reading of the ultrasound sensor is subtracted from the distance of the sensor from the stream bed. Determining this distance is straight-forward since the streams are ephemeral and remain void of water for most of the year. The project team periodically updates this distance. It is important to note that the streams in which the sensors are deployed are highly unstructured. A non-zero water level can be a result of localized ponding immediately beneath the sensor rather than actual flow. Furthermore, since the streams are also unregulated, the cross section at the observation points are also subject to change due to different human activities such as sand excavation, occasional off-road vehicles, or even human foot-traffic that can alter the stream bed. Therefore, stream levels should only be interpreted contextually with rainfall data. For the sensor installed at the dam, the overflow level, established at 1175 ft from sea level, is a reference point to determine the sensor height at its installation point. It is noteworthy that our dam station was installed at two different heights: from the installation date until July 5, 2021, the station height was 1187.33 ft from sea level, after which it was lowered to 1182.83 ft. The reported range in feet is subtracted from these sensor heights to determine the Lake Level.

Following the initial data processing pipeline, thorough data visualization is employed as a final quality control step. Visual inspection allows for the detection of potential anomalies or deviations from expected trends within the data that escape the automated quality checks. Any unexpected trends that are identified trigger further investigation to determine their root cause and ensure the data’s integrity. Through the advent of data-based methods and affordable computing at scale, a wealth of literature has emerged on the quality control of automatic weather stations that unlock new possibilities in ensuring the integrity of hydrometeorological databases^13,43–46^.

### Data upscaling

Following the detailed pre-processing and quality control measures outlined in the Data Processing section, we perform temporal upscaling to generate hourly, daily, and monthly datasets from the original 10-minute resolution data captured by our monitoring devices. The relevant WMO publications^34,47,48^ discuss data reduction guidelines for averaging, accumulation, statistical extremes, etc. This section describes the specific upscaling methods we employed for each variable.

#### Rain

For Hourly Rainfall, the data availability within each hour is assessed initially. If at least 60% of the 10-minute data in that hour is available, then the mean rainfall amount is calculated and multiplied by 6 to account for the 6 readings per hour. So, the unit of hourly precipitation data is millimeters per hour. Any hour with less than 60% data availability is marked as missing. For Daily Rainfall, the devices automatically accumulate and reset rainfall data at midnight, effectively providing the daily total. Therefore, the maximum value recorded during the midnight hour is retained for daily rainfall. The unit of the daily rainfall is millimeters per day. In the case of Monthly Precipitation, daily precipitation data is used, and a 60% data availability check is applied to convert the data into millimeters per month (cumulative).

#### Lake and stream water level

Upscaling for hourly, daily, and monthly lake levels utilizes the median of all valid data points within the respective time window. This approach prioritizes capturing the central tendency of the data, even with limited readings, as a single valid measurement within the period provides valuable information about the lake level. Similar to lake level, hourly, daily, and monthly stream level upscaling employs the maximum value from all valid readings within the specified time window. This method ensures capturing the highest stream level reached during the period, which is particularly relevant for monitoring potential flooding events.

## Data Records

The dataset is available on Figshare^49^ and comprises four Excel (.xlsx) files: **Namal-Catchment-Rainfall-Data.xlsx, Namal-Catchment-Stream-Levels-Data.xlsx, Namal-Catchment-Lake-Level-Data.xlsx**, and **Network Metadata - Namal.xlsx**. Each of the first three files contains multiple sheets organized by temporal resolution—10-minute, hourly, daily, and monthly. The sheet names follow the format “Temporal Resolution - Variable Name,” reflecting the frequency and type of data recorded. The accompanying metadata file provides essential context, including details of each monitoring station, variable descriptions, units of measurement, and explanatory notes.

Each sheet is structured to facilitate comprehensive data analysis. The top row lists the **station names**, acting as column headers, followed by the second row indicating the **latitude** coordinates, the third row showing the **longitude** coordinates, and the fourth row specifying the **units of measurement**. The subsequent rows contain the actual data, with the first column dedicated to timestamps. The timestamp format varies according to the data resolution: **YYYY-MM-DD HH:MM:SS** for 10-minute and hourly data, **YYYY-MM-DD** for daily data, and **YYYY-MM** for monthly data.

The dataset spans from **Nov-2020 to Sep-2024**, providing a record of meteorological and hydrological variables over this period. The coverage duration varies slightly between variables and temporal resolutions due to differences in data availability.

## Technical Validation

The validation process involved multiple steps to ensure the accuracy and reliability of the collected data. The calibration of the tipping bucket precipitation measurement system was conducted in two phases. The first phase established a conversion factor relating ticks to millimeters of precipitation. The second phase involved a comparative analysis with a calibrated beaker to validate the derived conversion factor. This validation process demonstrated consistent results between the two independent measurement methods, affirming the reliability of the conversion factor. The validation of the Ultrasonic Sonar in the monitoring network was carried out by taking manual range measurements using a measuring tape and comparing them with the sensor data. By conducting multiple measurements under various conditions, we ensured that the sensor readings were consistently accurate. The consistency between the manual measurements and the sensor data was observed to assess the reliability of the sonar readings.

**Fig. 4:**
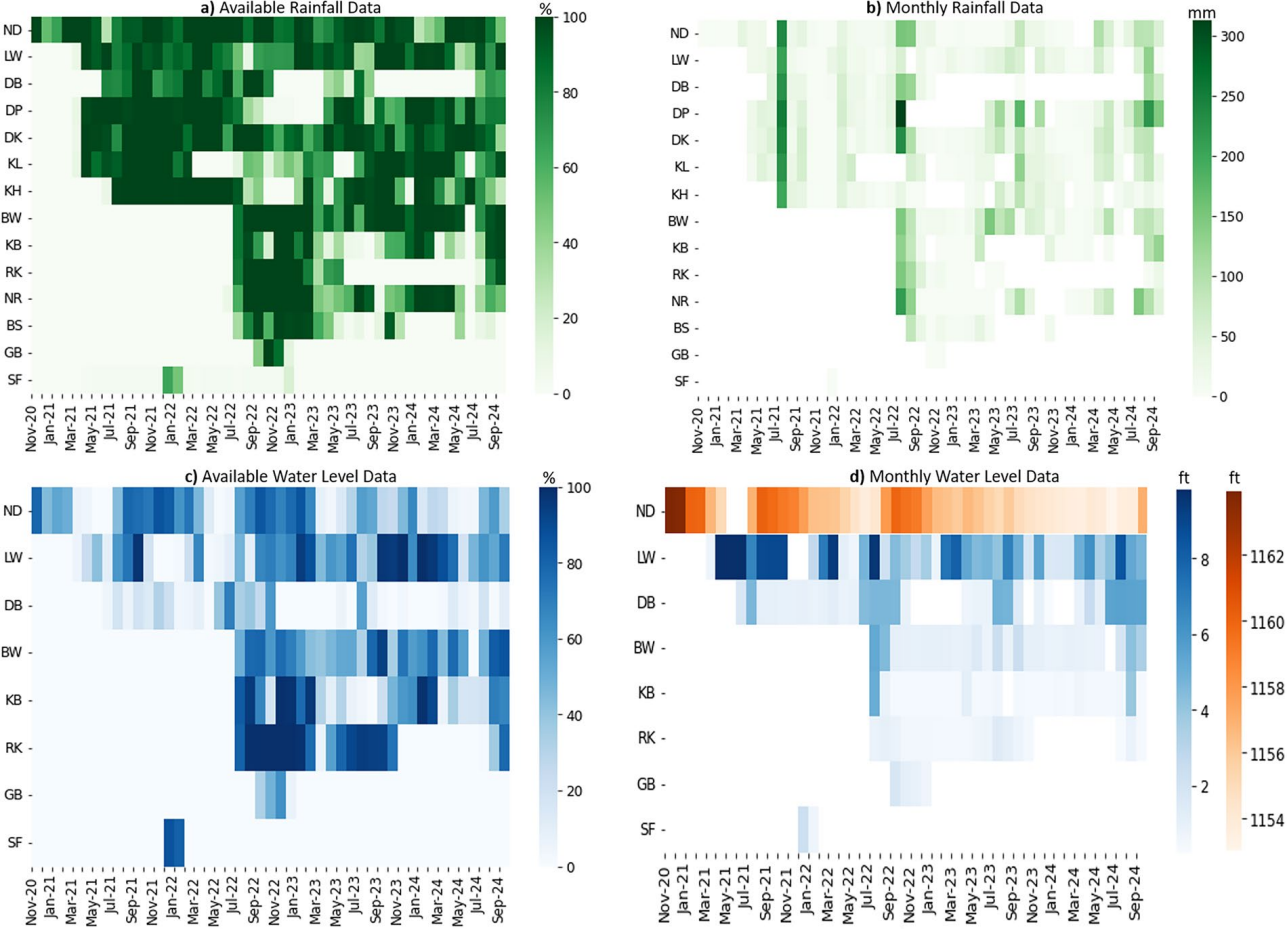
Heatmaps showing percentages of monthly data availability calculated based on 10-minute temporal resolution and also presenting the monthly averaged rainfall (mm) and maximum water levels recorded in each month during the study period.

To assess data quality and completeness, we conducted a detailed examination of data availability across all stations. Heatmaps (Fig. 4a,c) visualize the percentage of available data for rainfall and water level measurements, respectively, based on the 10-minute resolution dataset. These monthly summaries help identify periods of reduced data coverage, which may be attributed to either environmental conditions or operational factors. For example, Rikhi (RK) and Dhibba (DB) stations show a decrease in rainfall data availability starting from mid-2023. This reduction aligns with known cases of device tampering, which is a recognized risk given the public exposure of the sensors. In the case of water level data, we see that the proportion of missing values is significantly higher in the dry months than in the wet monsoon months. During dry periods, streams remain empty, and the unstructured streambeds limit the sonar sensors’ ability to detect a measurable surface. Such durations can be identified by matching the availability of rain data to the availability of water level data for each observation point. If rain data is available, but level data is not, it indicates missed readings due to the dry streambed and not sensor malfunctions. Under these conditions, missing readings may be interpreted as zero water level. For the Namal Dam (ND) station, the missing values in the dry season correspond to the water level going beyond the sensor’s range of measurement. It is also important to note that periods of missing values encountered due to sensor malfunction are still present (such durations of significant length are mentioned separately in the Data Gaps column in Table 2). Lastly, as mentioned in Table 2, the Saiflan (SF) station corresponding to the dam spillway was deployed only for short durations (due to logistical reasons) to capture the planned gate releases in the winter season. For the rest of the year, the spillway is dry. The extensive proportion of missing data for this observation point is thus attributable to the intermittent deployment of this station and not sensor failure. However, we still include the available data for spillway water levels, since it can provide valuable information in calibrating hydrological models of the reservoir, even though this observation point is not strictly part of the Namal Lake watershed.

We also examined station-level rainfall and water level data (Fig. 4b,d) for consistency with expected monsoonal patterns, and found them to align well with seasonal expectations. Maximum rainfall and corresponding stream and lake level peaks were observed during the monsoon months, supporting the physical plausibility of the measurements. This alignment with known hydrological patterns further validates the dataset’s integrity.

**Fig. 5:**
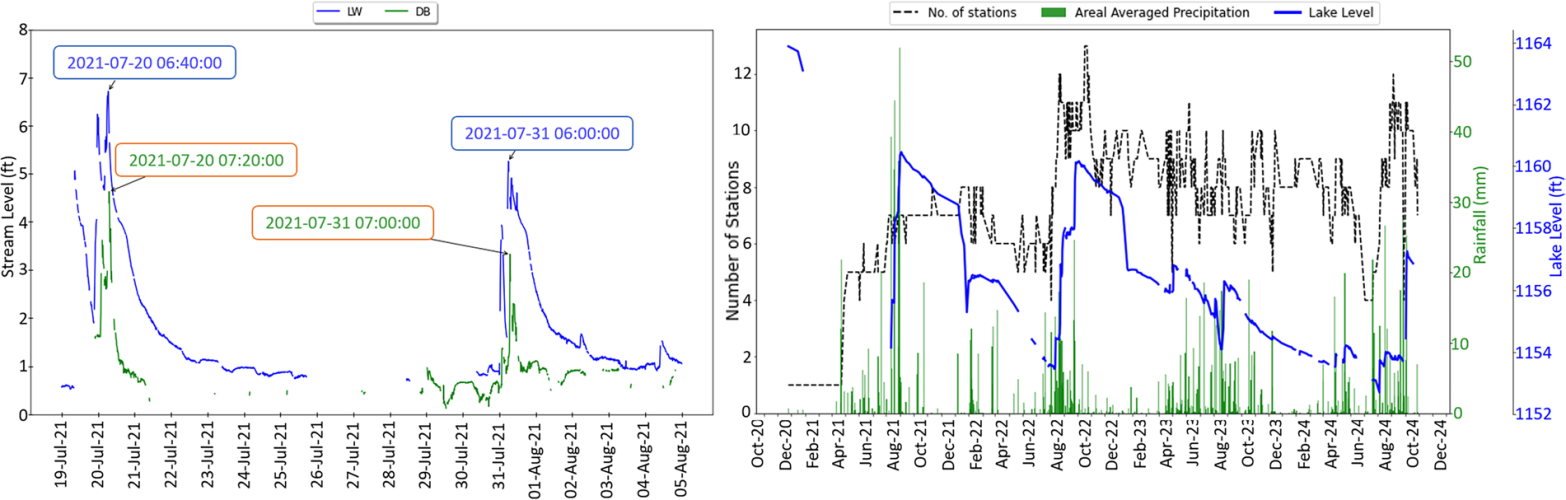
This figure represents the lag time between Lawa (LW) and Dhibba (DB) stations on the left, and the right figure presents the daily averaged rainfall, daily median lake level and the no. of stations based on which the rainfall averaged is being calculated.

Figure 5 (left) illustrates how variations in stream levels at the Lawa(LW) and Dhibba(DB) stations reflect the dynamic flow behavior during rainfall events. Observed lag times, such as 40 minutes on July 20 and around 1 hour on July 30, demonstrate the consistency of the measurements and the network’s ability to capture real hydrological responses. Additionally, data coverage on rainy days was assessed in conjunction with daily changes in lake levels (Fig. 5, right). This analysis ensured that sufficient spatial data was available to compute areal averages using geostatistical methods such as Ordinary Kriging^50^, confirming the reliability of the dataset for spatiotemporal analyses.

## Usage Notes

This dataset presents a valuable resource for researchers studying the hydrology of mountainous lake ecosystems. This high-resolution (10-minute) *in-situ* dataset, encompassing rainfall, stream inflow depths (ft), and lake water levels from 14 locations within Pakistan’s Namal Lake catchment area, enables diverse hydrological research applications at the catchment level. The detailed temporal resolution allows for capturing the dynamic behavior of water flow within the catchment, ideal for calibrating and validating hydrological models. Predictive modeling using this data can help forecast lake levels, streamflow patterns, and potential flood risks, contributing to early warning systems and disaster preparedness measures. Information on lake water levels and stream inflows is crucial for effective water resource management, enabling users to optimize water allocation strategies and assess water availability. To preserve data integrity, missing values due to sensor malfunctions or communication issues are retained across all resolutions (10 minutes, hourly, daily, and monthly). Users are encouraged to address these gaps using appropriate techniques aligned with their specific analyses.

## Data Availability

The custom code used for data processing in this study, including kriging for precipitation averaging, quality control, and upscaling to different sampling rates, is publicly available on GitHub at https://github.com/LUMS-WIT/Hydro_met_net_Namal. The kriging algorithm for spatial interpolation and precipitation averaging was implemented in MATLAB (R2023a). Quality control tasks, such as data cleaning, missing value handling, and unit conversions, and the upscaling tasks were performed using Python.
